# Alcohol industry involvement in the delayed South Africa Draft Liquor Amendment Bill 2016: a case study based on freedom of information requests

**DOI:** 10.1186/s12992-025-01097-5

**Published:** 2025-03-25

**Authors:** Gemma Mitchell, Pfumelani Siwela, Susan Goldstein, Aadielah Maker Diedericks

**Affiliations:** 1 Institute of Social Marketing and Health, Faculty of Health Sciences and Sport, University of Stirling, Stirling, UK; 2 Southern African Alcohol Policy Alliance, Cape Town, South Africa; 3 SAMRC/WITS Centre for Health Economics and Decision Science; PRICELESS SA, School of Public Health, Faculty of Health Sciences, University of the Witwatersrand, Johannesburg, South Africa

**Keywords:** Alcohol industry, Alcohol policy, South Africa, Corporate political activity, Policy formulation, Commercial determinants of health

## Abstract

**Background:**

South Africa is reported to have one of the highest per capita rates of alcohol consumption among drinkers globally, with alcohol harms exacerbating socio-economic inequalities in the country. The Draft Liquor Amendment Bill 2016 proposed new restrictions on alcohol advertising, availability, and liability of retailers and manufacturers for harm related to any contravention of the regulations. To date, the Bill has not progressed through the legislative process. The alcohol industry is known to use a diverse set of strategies to delay evidence-based policies globally.

**Methods:**

We aimed to explore Bill-related activity by industry within the National Economic and Development Labour Council, a multi-stakeholder forum that assesses socio-economic policies before they reach parliament. On 06 July 2023 we made a Request for Access to Record, using form two of the Promotion of Access to Information Act (PAIA), no. 2 of 2000 to the National Economic and Development Labour Council for access to minutes of all meetings, reports, and any other publications related to the Bill between January 2016 and December 2022. Informed by Ulucanlar et al’s (2023) model and taxonomies of corporate political activity, we extracted data on industry Bill-related activity and thematically analysed key events, presented here as a narrative synthesis.

**Results:**

We identified activity by 14 alcohol industry organisations related to the Bill between 2016 and 2022. Industry representation on five National Economic and Development Labour Council-related committees identified between 2017 and 2021 facilitated their involvement in Bill-related discussions and supported access to other government departments. Community representation was low in all committees compared to industry, labour, and government. Industry funded two socio-economic assessments of the Bill in 2017 and 2022, despite an independent socio-economic impact assessment having already been completed. The 2017 report delayed progress of the Bill, and the 2022 ‘re-evaluation’ was more critical of the proposed measures, with the differing conclusions attributed to different methodologies. During the covid-19 pandemic, industry used a ‘carrot and stick’ approach of legal threats and donations to attempt to move towards self-regulation via a social compact. The National Economic and Development Labour Council confirmed in 2023 that the social compact was unsuccessful.

**Conclusions:**

Early ‘regulatory capture’ gave the alcohol industry the opportunity to shape assessment of the Bill within the National Economic and Development Labour Council. Our findings are in line with previous studies on corporate influence on policy globally, and support calls for a reassessment of the role and proportion of industry representation within the National Economic and Development Labour Council locally.

## Introduction

Sub-Saharan Africa has been identified as a key market for growth for the alcohol industry due to high abstention rates across most regions, high population growth and urbanisation, and weak uptake of World Health Organization (WHO) ‘best buy’ alcohol control policies [1–3]. Global retail alcohol sales in 2017 were estimated to be over $1.5 trillion, with sales and profits concentrated in a small number of transnational companies [4]. The alcohol industry, like other health-harming industries such as tobacco, ultra-processed foods, and gambling, uses their extensive resources to oppose evidence-based policies to address the harms of their products, and promote self-regulatory options which support commercial interests but have little or no benefit for public health [5–9]. Although research on alcohol industry activity in sub-Saharan Africa has increased in recent years [for example, 10, 11–14], there remains a lack of research on commercial involvement in health policy in low and middle-income countries (LMICs), particularly in relation to the policy formulation process [10].

South Africa is reported to have one of the highest per capita rates of alcohol consumption among drinkers globally [15], with 62,000 adults dying from alcohol-attributable causes in 2015 [16]. This is despite high rates of abstention– in 2016, only approximately 20% of female and 50% of males were current drinkers [17]. Heavy episodic drinking is therefore a major problem, with a prevalence rate of 74% reported among male drinkers aged 15–19 in 2016 [15]. South Africa has been described as the most economically unequal country globally [18], with alcohol harms exacerbating the problem. A modelling study of alcohol-attributable mortality found 60% of all alcohol-attributable deaths occurred in the lower 30% of social strata [19]. Although the alcohol industry makes a significant contribution to the domestic economy via employment, output, and export earnings, the cost of alcohol harms has been estimated at -10-12% of the 2009 gross domestic product [20].

Alcohol is currently regulated in South Africa under the Liquor Act No. 59 of 2003 [21]. Any proposed changes to this and other policies are assessed by the National Economic Development and Labour Council (NEDLAC), a statutory body that is described as ‘the vehicle by which government, labour, business, and community organisations seek to cooperate, through problem-solving and negotiation, on economic, labour and development issues, and related challenges facing the country’ [22]. The most recent attempt to update the legalisation is the Draft Liquor Amendment Bill 2016 (hereafter referred to as the Bill), which proposed new restrictions on alcohol advertising, availability, and liability of retailers and manufacturers for harm [23]. The changes include alcohol outlets not being allowed within 500m of schools among other public places; increasing the legal drinking age from 18 to 21; advertising restrictions on print media, television, radio, billboards, print media and other public areas except at site of sale; and placing liability on alcohol retailers and manufacturers for harm related to any contravention of the legislation. The Bill was gazetted (shared) for public comment in 2016. In 2017, an amended version of the draft Bill was circulated but not made publicly available. There were differences in this version; for example, proximity restrictions for the retail of alcohol within 500m of public places such as schools, places of worship, and residential areas were removed in the 2017 version, and the number of proximity restrictions for manufacturers and distributors were reduced [24]. Restrictions on marketing and advertising, raising the legal drinking age, and restrictions on distribution and supply to unlicensed premises remained the same [24]. Unless otherwise stated, we refer to the 2016 official, published version throughout. At time of writing the Bill has not progressed through the legislative process.

If implemented in full, the Bill could significantly reduce alcohol harms in South Africa. The Bill includes two of the WHO ‘best buy’ alcohol control policies (bans or comprehensive restrictions on alcohol; restrictions on alcohol availability) [15]. When the Russian Federation applied these policies, plus taxation and price increases, there was a 43% reduction in alcohol consumption between 2003 and 2016 [25]. This supported a drop in all-cause mortality of 39% in men and 36% in women between 2003 and 2018 [25]. There are few policy process studies in LMICs, in particular on policy formulation [10, 26]. A previous study shows that the alcohol industry was a key actor involved in the 2013 Draft Control of Marketing of Alcoholic Beverages Bill, which has also not been implemented [10]. Focusing on policy formulation can provide insight into strategies used by powerful industries in LMICs to prevent progress towards the adoption and implementation of evidence-based policies [10, 26]. Media and advocacy groups’ heavy criticism of an industry-funded report on the relationship between trauma admissions and temporary alcohol sales bans during the Covid-19 pandemic [27, 28], combined with NEDLAC’s central role in assessing proposed legislation before it is introduced in parliament, led us to explore the extent of alcohol industry involvement in NEDLAC activity related to the Bill. Here, we provide a critical analysis of industry Bill-related activity within NEDLAC. Analysis of the wider policy formulation process related to the Bill is beyond the scope of the current study.

## Methods

### Design

We used an exploratory case study methodology to critically analyse alcohol industry activity within NEDLAC related to the Draft Liquor Amendment Bill 2016. The case study approach is useful when there is a need to obtain an in-depth appreciation of an issue, event or phenomenon of interest in its real-life context [29]. Critical social research acknowledges and centres the interrelationship between data, theory, pre-existing knowledge, and socio-political context [30] and has been used in previous studies of the relationship between governments and industry [31, 32]. We explore the extent to which alcohol industry activity within NEDLAC influenced progress of the Bill.

Freedom of information laws have been described as underutilised research tools [33] that are particularly helpful when studying public-private partnerships or relationships [31, 34]. Two objectives of the South Africa Promotion of Access to Information Act (PAIA), no.2 of 2000 are to ‘promote transparency, accountability and effective governance of all public and private bodies [and] to assist members of the public to effectively scrutinize and participate in decision making by public bodies’ [35]. Data received via such requests are by nature incomplete; as in previous studies using this method, we therefore supplemented these data with other publicly available information and published literature where possible [31, 36].

### Data collection

On 06 July 2023 we made a Request for Access to Record, using Form 2 of the Promotion of Access to Information Act (PAIA), no. 2 of 2000 [35] to NEDLAC for access to minutes of all meetings including plenary sessions, working group meetings, task team meetings and sub-committee meetings, reports, studies, research papers, and any other publications commissioned or produced by NEDLAC on topics related to the Bill between January 2016 and December 2022. We also requested agreements, memoranda of understanding, and any other forms of formal agreements entered by NEDLAC regarding the alcohol industry and correspondence, including letters, emails, faxes, and memoranda exchanged between NEDLAC and relevant stakeholders, such as government departments, trade unions, industry associations, and civil society organizations in relation to the Bill within those same dates. We received 56 documents from NEDLAC totalling 573 pages. These included meeting minutes, draft and final reports, letters, PowerPoint presentation slides, email correspondence, industry guidelines, and a webpage. We received only five documents related to 2017, and no documents covering 2018, 2019, or Jan-Jun 2020. Subsequently, on 23 November 2023 we made a second request to NEDLAC for documents during that period but received duplicates of documents previously sent and no new documents.

### Data analysis

We drew on the READ approach [1] Ready materials, 2) Extract data, 3) Analyse data; and 4) Distil findings) [37] to help organise the data, creating a Microsoft Excel file to screen the documents for duplicates and identify industry actors involved. Excluding duplicates led to a dataset of 43 documents totalling 477 pages. A large proportion of the data covered the second half of 2020 and the Covid-19 pandemic response.

The Ulucanlar et al. (2023) model and taxonomies of corporate political activity [5] outline both action and framing strategies that health-harming industries use to influence policy. The framing taxonomy outlines three frames (the policy actor; problems; and solutions), and the action strategy taxonomy outlines six actions (access and influence policy-making; use the law; manufacture support for industry; shape evidence to manufacture doubt; displace and usurp public health; and manage reputations to industry’s advantage) used by health-harming industries globally to advance their interests [5]. Using this model, we focused on action strategies as this dataset gives insights into actions that may not be possible via other methods. We extracted all data on alcohol industry actors involved, the non-industry actors they interacted with, and dates and content of all events referred to within the dataset. We summarised each event, and subsequently identified key industry involvement across a timeline of Bill-related activity which we present here in a narrative synthesis [38]. Data extraction was independently conducted by two authors, and key events were coded by one author and reviewed by a second author. Data extraction from scientific reports was performed separately; due to resource limitations, we extracted conclusions and industry activity only from the reports. Disagreements about what should be included as key events and how they were coded were resolved as a team. An additional framing analysis of the NEDLAC report on the Bill, which contained detailed industry perspectives [39] was conducted by one author and reviewed by a second author.

Co-author expertise on alcohol policy development in South Africa informed how the received dataset was verified and supplemented by other publicly available information. This included the NEDLAC website and annual reports, media reports, industry websites, and industry-funded reports. In line with previous studies using equivalent freedom of information requests [31, 40, 41], as data were in the public domain and there were no human participants, the study did not require ethical approval. Industry was referred to as ‘business’ throughout the dataset. For clarity, we use ‘industry’ to refer to the alcohol industry, except for quotes where the original use of ‘business’ is retained.

## Results

We identified 14 alcohol industry groups in the dataset (see Table 1). Alongside industry membership of the NEDLAC Executive Council [42] and Trade and Industry Chamber [43], industry representation on five committees facilitated their involvement in Bill-related discussions (see Table 1). Much of the data relates to 2017, when a NEDLAC report on the Bill was produced [39] and 2020/21, when the pandemic response and policy in the form of a social compact appeared to take precedence over the Bill. We outline the mechanisms of contact between industry and other stakeholders regarding the Bill, industry activity related to the Bill during the Covid-19 pandemic, and the use of science by industry below.

**Table 1:** Key alcohol industry organisations identified in the dataset

Alcohol industry actor	Example activity
Transnational alcohol companies	
AB InBev	NEDLAC Liquor Task Team member. Named party to a July 2020 version of a draft social compact* (as part of Beer Association of South Africa). See also South African Breweries
Diageo	Named party to a July 2020 version of a draft social compact (as part of South African Liquor Brand Owners Association)
Heineken	NEDLAC Liquor Task Team member. Named party to a July 2020 version of a draft social compact (as part of Beer Association of South Africa). See also Distell
Pernod Ricard	Named party to a July 2020 version of a draft social compact (as part of South African Liquor Brand Owners Association)
Subsidiaries of transnational companies	
Distell (acquired by Heineken in 2023)	NEDLAC Liquor Task Team member. Named party to a July 2020 version of a draft social compact (as part of South African Liquor Brand Owners Association)
South African Breweries (subsidiary of AB InBev since 2016)	In August 2020, government noted that: ‘there’s been a threat from SAB [South African Breweries] to withdraw the 2.5 million that they were going to invest in the sector on the basis of the fact that they were not able to trade’. Meeting minutes report that industry declined to rescind the withdrawal of the funds (NRRTT on the liquor industry meeting minutes, 24th August 2020). Named party to a July 2020 version of a draft social compact (as part of Beer Association of South Africa)
Social Aspects and Public Relations Organisations (SAPROs)	
The Association for Alcohol Responsibility and Education (Aware); formerly known as the Association for Responsible Alcohol Use (ARA) *(funded by the alcohol industry* [44]. *Includes Diageo and Heineken board members)* [45].	During discussion of the social compact, industry representatives proposed self-regulation measures could be implemented through Aware. Presented to NEDLAC IN February 2021 on the industry-wide response to the pandemic, including reported donations to the pandemic response
Trade associations	
Beer Association of South Africa (BASA) *(represents Craft Brewers Association South Africa*,* Heineken South Africa*,* South African Breweries and United National Breweries*) [46]	NEDLAC Liquor Task Team member. Named party to a July 2020 version of a draft social compact. One of three industry funders of a 2022 socio-economic assessment of the Bill [47]
National Liquor Traders Association (NLTA) *(including/also known as Liquor Traders Association of South Africa (LTASA) National Liquor Traders Council (NLTC) and South African Liquor Traders Association (SALTA))*	NEDLAC Liquor Task Team member. Named party to a July 2020 version of a draft social compact
South African Liquor Brand Owners Association (SALBA) *(AWARE board member; Diageo and Heineken are executive members)* [48]	NEDLAC Liquor Task Team member. One of three industry funders of a 2022 socio-economic assessment of the Bill [47]
Vinpro (wine industry)	Named party to a July 2020 version of a draft social compact. One of three industry funders of a 2022 socio-economic assessment of the Bill [47]
Wider industry groups	
Agricultural Business Chamber South Africa (Agbiz) *(lists South Africa Wine as members)* [49]	NEDLAC Liquor Task Team member
Business Unity South Africa (BUSA) (includes South African Breweries as members [50])	Apex body ‘formally recognised representative of business at NEDLAC’ [51]. NEDLAC Liquor Task Team member. Commissioned a 2022 socio-economic assessment of the Bill [47]
Consumer Goods Council of South Africa (CGCSA) (membership includes retailers and other industries including tobacco [52]	NEDLAC Liquor Task Team member. Named party to a July 2020 version of a draft social compact

*The social compact refers to efforts by the South African government ‘to forge a comprehensive social compact that would join all social partners in a common programme to rebuild the South African economy [post-Covid-19 pandemic] and enable higher growth’ [53]

### Mechanisms of contact between industry, government, and other stakeholders

We identified five committees set up between 2017 and 2021 that facilitated or proposed to facilitate contact between industry, government, community, and labour representatives (see Table 2). Notably, industry representation was far higher than community representation in all committees where this information was available. Committee membership supported contact between industry and senior government officials during the pandemic. For example, the Minister of Health attended a NEDLAC meeting in December 2020, during which industry presented on self-regulatory measures in place to prevent a second wave of Covid-19. Even when industry representatives were not present, their role in the committees meant their input could be shared with senior government officials. For example, proposed measures to avoid a resurgence of Covid-19 were planned to be used as a basis for a National Joint Operational and Intelligence Structure (NATJOINTS) meeting in December 2020. A planned government meeting with the President in August 2020 was also discussed by the NEDLAC rapid response task team (NRRTT), including to develop proposals to bring to that meeting, although there is no evidence that alcohol industry representatives attended. A meeting between the Minister of the Department of Trade, Industry and Competition (DTIC) and the NEDLAC rapid response task team was planned in July 2021 to ‘hear the views of the social partners on the liquor restrictions’, although it is not known if this went ahead.

**Table 2:** Membership and activity of NEDLAC committees

Committee and years of key activity	Reported membership						Nature of key activity
	Industry (most often referred to as ‘business’ in the dataset)	Government			Labour	Community	
NEDLAC Trade and Industry Chamber Liquor Amendment Bill Task Team; 2017 (39)	7	3			5	None noted	The Department of Trade and Industry tabled the National Liquor Amendment Bill to the task team for engagement in April 2017. The task team submitted the NEDLAC report on the Bill, which was based on detailed review by all parties to identify areas of agreement and disagreement between April and November 2017 (39)
NEDLAC Trade and Industry Chamber 1-aside Task Team; 2017 (39)	Sub-committee of the above (individual members unknown)						Set up to support the commissioning of industry-funded research on the economic impact of the Bill (39 pp.1–2),
NEDLAC Rapid Response Task Team (NRRTT) Liquor Industry Sub-Committee; 2020; 2021 (54)	15 reported in (54) (pp.56 − 7), although 24 reported to attend at least one meeting in July 2020		8	7		1	In 2020, a NRRTT was set up as part of the wider government response to the Covid-19 pandemic. A sub-committee was created to address the impact of the temporary alcohol sales bans on the industry (54)
Liquor Advisory Council; 2020	Yes (number unknown)		Unclear	Unclear		Unclear	In November 2020, industry confirmed it had set up the council, apparently to discuss their proposals for the social compact
NEDLAC Rapid Response Liquor Industry 2-aside Task Team; 2021	3		7 (including 5 NEDLAC officials)	2		1	In June 2021, another NRRTT sub-committee was set up, which included discussion of the social compact

### Industry shaping evidence to manufacture doubt

We identified two industry-funded assessments of the Bill, which were completed in 2017 and 2022 (see Table 3). This is in addition to the (previously reported) industry-funded research reports produced during the pandemic [55], when a social compact appeared to take precedence over the Bill.

**Table 3:** Industry-linked reports on the Draft Liquor Amendment Bill 2016

Title of report	Month/ year	Funder information	Authors	Key conclusions
‘Evaluating the economic, health and social impacts of the proposed Liquor Amendment Bill, 2017’ (56))	Oct 2017	‘The study is commissioned by NEDLAC and is funded by the South African Liquor Brands Association (SALBA)’ (56 p10)	Genesis Analytics (consultancy firm)	Report estimates following impacts of the Bill: 185 lives a year saved due to a 3% reduction in alcohol-attributable road traffic fatalities; reduced alcohol consumption; minimal impact on employment for alcohol industry; reduced competition in industry; and ‘the policy changes are likely to reduce public health costs’ (56 pp.3–6)
‘An independent re-evaluation of the economic, health and social impacts of the Liquor Amendment Bill, 2016’ (47)	Sep 2022	‘This work is commissioned by Business Unity South Africa (BUSA) and is funded by the South African Liquor Brands Association, VINPRO and the Beer Association of South Africa’ (47 p.8)	Genesis Analytics (consultancy firm)	Implementing an increase in the legal drinking age from 18 to 21 and above-the-line advertising restrictions was estimated to lead to a drop of between 3.51–4.44% in aggregate consumption and save 376 lives through a reduction in alcohol-attributable road traffic fatalities (47 p.4). Time and zoning restrictions on the manufacture and distribution of alcohol and extended legal liability for harm arising from selling to unlicensed retailers ‘will have no discernible impact on aggregate consumption or on social harms, while potentially creating economic cost for industry’ (47 p.4)

The 160-page report evaluating the potential impact of the Bill in 2017 [56] was a response to a Department of Trade and Industry (DTI) socio-economic impact assessment (SEIAS) of the Bill (39 p1). Since 2015, all draft policies, Bills or regulations in South Africa must have a SEIAS [57], and the Department of Planning, Monitoring and Evaluation [DPME] granted approval for the Bill to proceed based on a completed SEIAS in June 2017 (39 p1). Yet, the NEDLAC task team concluded this was insufficient:

Although the SEIAS presented before the Task Team met the required guidelines of DPME and received the certification, the stakeholders at the NEDLAC Task Team were of the view that the SEIAS did not quantify job losses. While the parties agreed that the SEIAs document would not be replaced, it was agreed that NEDLAC would conduct the research to inform the deliberations of the NEDLAC constituencies.

(NEDLAC report on the Liquor Amendment Bill, November 2017, (39 pp.1–2))

When government sought to clarify the purpose of the additional research:

Business stated that the outcome of the research would better inform the Social Partners on the impact of the Bill and what could be done to mitigate the unintended consequences hence Business had indicated that it was willing to fund the research. Business reiterated that this would not be a [sic] Business research but a NEDLAC research funded by Business.

(Liquor Amendment Bill Task Team Meeting, 20th July 2017)

Thus, an extra impact assessment was commissioned by NEDLAC and funded by the alcohol industry [39]. Industry suggested the choice of consultancy firm:

Genesis Analytics was appointed on the 31st [of] August 2017 after Business informed the 1-aside team that Genesis analytic will be able to conduct the research study within the time constraint.

(NEDLAC report on the Liquor Amendment Bill, November 2017 (39 p.2).

Completion of this extra assessment caused at least a one-month delay to the NEDLAC report on the Bill and was not available for the task team to consider during their deliberations (39 p.2). The report concluded that “the WHO guidelines should be adapted to South Africa”, including applying zoning restrictions to alcohol-licensed premises, restrictions on marketing, and a staggered introduction of an increased legal drinking age (56 p.135).

The 184 page ‘re-evaluation’ of the policies proposed in the Bill in 2022 by the same consultancy firm included many of the same authors of the 2017 report. The authors came to a similar conclusion regarding aggregate consumption, yet were more critical of the Bill overall [47]. Implementing an increase in the legal drinking age from 18 to 21 and above-the-line advertising restrictions was estimated to lead to a drop of between 3.51 and 4.44% in aggregate consumption (47 p.3) and save 376 lives (double that of the 2017 estimate). Yet, the report describes these interventions (applied together) as “one of relatively low social and health benefits, but equally, relatively low economic cost” (47 p.3). Notably, the number of lives saved was based on a reduction in road traffic incidents only rather than the much broader range of alcohol harms, with the authors stating:

While some of the likely reduction in consumption as a result of the proposed amendments could relate to hazardous consumption (such as youth consumption), the study does not quantify the extent of the change in hazardous consumption in particular, as it is difficult to assess which groups of drinkers will be impacted by the interventions.

(Genesis Analytics evaluation of the Liquor Amendment Bill, 29th September 2022 (47 p.4))

Regarding mandatory compliance with the Broad-based Black Economic Empowerment (B-BBEE) Act 53 of 2003^1^, the report made similar arguments to those made by the alcohol industry in the 2017 NEDLAC report on the Bill (see below), for example emphasising the impact on small, “family-owned” businesses and questioning the legality of the proposal (47 p.5). A page was dedicated to alcohol brands “increasingly using their far audience reach to share information about responsible drinking and pertinent community challenges” since 2020 (47 p.34). When assessing the economic costs of alcohol, the report included a heavily criticised, industry-funded analysis that the alcohol industry has a net positive impact on society. Although the report noted the industry funding, we could find no reference to the extensive criticisms the analysis had received [58]. Finally, when noting consultations with relevant groups, the report categorised Aware (an alcohol industry-funded [44] ‘social aspects and public relations organisation’) as a civil society actor, despite there being a separate section for industry (47 p.166).

The authors of the 2022 report acknowledged the different conclusions compared to their 2017 analysis, and described this as due to “a more thorough methodology”, including “discounting international evidence based on conduciveness parameters for regulation and enforcement in South Africa”, using more conservative estimates from consulted stakeholders, using updated estimates for general and youth population consumption, and more recent data on road traffic fatalities (47 p.161).

### Industry framing within the NEDLAC report on the Liquor Amendment Bill

NEDLAC, including government, labour and industry representatives, completed a review of the Bill between April - November 2017. The result was a report on the Bill [39], with NEDLAC membership facilitating detailed industry comment, including on implementation. No community involvement in the NEDLAC review was noted in the report, although community comment had been sought prior when the Bill was gazetted and “consideration of comment arising from public consultation was finalised in March 2017” (39 p.1). Industry framed an increased drinking age, and restrictions on advertising and alcohol outlet locations as unacceptable, ‘bad’ solutions to alcohol harms, despite such approaches being described by the WHO as the most cost-effective policy interventions for addressing alcohol harms [59]. Various industry arguments were made throughout the document that portrayed the alcohol industry as key economic actors who are socially responsible and concerned with social justice. This was combined with framing elements of the Bill as undesirable, raising concerns that (a) parts of the Bill would disproportionately impact small businesses and young entrepreneurs; and (b) parts of the Bill may not align with the Broad-based Black Economic Empowerment (B-BBEE) Act 53 of 2003. At times, industry arguments appeared contradictory. For example, when opposing social media marketing restrictions, industry stated “the ‘age gate controls’ that exist can and should be strengthened to ensure that under 18s do not access liquor adverts’, yet in the next sentence stated “controlling the internet is impossible” (39 p.27).

Industry suggested alternative proposals focused on self-regulation and voluntary action by corporations, and appeared to suggest the building of schools should work around existing alcohol outlets, instead of closing alcohol outlets near schools. There appeared to be more agreement between the parties regarding the functions of a new National Liquor Regulator; proposals to regulate specific trading days and hours for alcohol to be distributed and manufactured, and measures to address production of counterfeit products. A summary of the key framing strategies used by industry in the report is provided in Table 4.

**Table 4:** Key framing strategies used by industry within the NEDLAC report on the Draft Liquor Amendment Bill (2017)

Proposal in the Draft Liquor Amendment Bill (including amendments proposed post-sharing the Bill for public comment)	Industry frame-supporting claims	Illustrative examples (all quotes from [39])
The unacceptable, ‘bad’ solution		
Increase legal drinking age from 18 to 21 years	Proposal contravenes existing norms, rules and laws	“The provision [is] incongruent with progressive constitution and the age of majority act. A 19-year-old may marry without the consent of his or her parents but would need his parents’ consent to celebrate the occasion with a glass of sparkling wine” (p.29)
		“Under the current definition of a minor, the law treats under 18’s as minor’s who are usually processed in a juvenile court. Persons over the age of 18 are treated as adults and are prosecuted as such. Consumers who are between 18 and 21 who lie about their age will be prosecuted as adults and we are therefore creating a new category of criminals” (pp.29–30)
		“A socio-economic impact assessment must be conducted on the implications for university campuses, especially considering that students are of a legal drinking age under the current definition” (p.25)
“’Educational institution’ means a place where people of different age attend to gain knowledge and education which includes, private and public institutions, childcare, preschools, elementary schools, high schools and institutions of higher learning” (p.25) (changed from ‘school’ used in earlier draft of the Bill)	Proposal contravenes existing norms, rules and laws	“Many universities prefer students to use the bars and pubs on campus because it means that students do not have to drive or walk back to campus. A socio-economic impact assessment must be conducted on the implications for university campuses, especially considering that students are of a legal drinking age under the current definition” (p.25).
Restrictions on advertising on billboards (including near educational institutions), pamphlets, internet, television and radio (beyond specific time slots) and cinemas and theatres	Proposals are unnecessary and unacceptable	“[Restrictions on billboards] will impact negatively on the entrepreneurs who are increasingly using billboards as a platform to enter the media industry’ (p.27)
	Proposals will lead to losses for business, economy and society	“The ‘age gate controls’ that exist can and should be strengthened to ensure that under 18s do not access liquor adverts” (p.27)
Restrictions on liquor outlet locations, including within a specific radius of schools (later educational institutions), residential areas, and places of worship and recreation	Proposals contravene norms, rules and laws	“This provision seeks to usurp the constitutional powers of the provincial authorities…this clause seeks to elevate the norms and standards to legislation” (p.32)
	Policy will fail and have perverse consequences	These outlets simply will pop up illegally elsewhere and will lead to the illegal trade in alcohol outside the regulators control” (pp.27 − 8)
	Proposals will lead to losses for businesses, economy and society	“This provision would essentially prohibit the creation of viable competitors to the incumbent players and severely limit the transformational agenda of government” (p.31)
Restrictions on advertising on billboards (including near educational institutions), pamphlets, internet, television and radio (beyond specific time slots) and cinemas and theatres Restrictions on liquor outlet locations, including within a specific radius of schools (later educational institutions), residential areas, and places of worship and recreation	Proposals contravene norms, rules and laws	“If the manufacturer and distributor can demonstrate compliance with the regulations, then unless the state is able to prove the contrary there should be no liability” (p.34)
Applicants to the National Liquor Regulator (to be registered as an alcohol manufacturer or distributor, or both) must meet the Broad-Based Black Economic Empowerment level of compliance	Proposal will lead to losses for businesses, economy and society	“Becoming compliant and remaining compliant [with the B-BBEE Act] is not as simple as suggested in the provisions of the bill. Businesses could fall 2 or 3 levels because black shareholders choose to sell shares in a business to a white investor. It would severely restrict the black entrepreneur’s right to sell his or her shares and make a profit from the sale if the seller has restrictions placed on him or her because the business wishes to retain its BBEEE score” (pp.30 − 1)
The acceptable, ‘good’ solution		
*In response to proposals to restrict advertising and increase legal drinking age from 18 to 21*:		
Self-regulatory marketing guidelines	Solutions should be self-regulatory and not disrupt business	“The Bill should apply the guidelines in the ARA [now Aware] code of commercial communication which govern alcohol advertising content and times. These codes represent global best practice” (p.26)
Education programmes		“Industry would make resources available to run educational programmes and conduct research” (p.29)
*In response to restrictions on liquor outlets located within a specific radius of schools (later educational institutions)*,* residential areas*,* and places of worship and recreation*:		
Education programmes	Solutions should target individuals, not whole populations	“Consumers [should] be held accountable for their purchasing behaviour. By law licensees are required to display their licenses on the premises. Government should educate consumers to report unlicensed businesses and how to identify them” (p.35)
*In response to restrictions on liquor outlets located within a specific radius of schools (later educational institutions)*,* residential areas*,* and places of worship and recreation*:		
No building of schools near licensed premises	Solutions should be self-regulatory and not disrupt business	“Business proposed that the current license holders should not lose their license to trade and that spatial planners for cities should plan ahead so that schools etc. are not built close to existing licenses in future” (p.32)
Education programmes	Solutions should target individuals, not whole populations	“Encouraging responsible consumption of alcohol and educating consumers will have a greater impact than closing outlets (p.32)

### Industry carrot and stick during the pandemic: donations, legal action and a failed social compact

During the pandemic, three temporary alcohol sales bans were implemented (March 27 to June 1, 2020; July 13 to August 17, 2020 and December 28, 2020 to February 2, 2021) with a view to freeing up health services to deal with Covid-19, which was successful [60]. The alcohol sales bans were discussed within NEDLAC, and in July 2020 the idea of a social compact was raised. Groups involved (industry, government, labour, and community representatives) were all referred to as social partners, and there were reports of discussions about the social compact between the Minister of Health and the NEDLAC executive council:

There was a [sic] further preparations to convene a meeting with the President in the afternoon and it was agreed that this matter [alcohol ban] should be on the agenda if there are solid proposals in place…Way forward: social partners to develop a social compact document that would be presented at the meeting [with the president] of 03 August 2020.

(NRRTT on the liquor industry meeting minutes, 27th July 2020)

Occasionally, the various litigation threats and court challenges by industry were referenced during these discussions. For example, in a NRTT on the liquor industry meeting on 27th July 2020, industry indicated that there would not be further legal action against the temporary sales bans. This was followed up by letter three days later to confirm:

We see no reason to engage in litigation while we are having constructive, meaningful and timely discussions with government on the steps necessary to deal with the Covid-19 pandemic.

(Letter to NEDLAC convenor from Vinpro, SALBA, BASA, NLTC and LTASA, 30th July 2020)

Other legal action by industry continued into 2022 [55, 61].

The aim of the social compact was reported as follows:

The social compact sought to ensure that the liquor industry committed to provide various kinds of support to the health sector as well as introduce harm reduction programmes in exchange for a [sic] lifting of restrictions.

(NEDLAC annual report 2020/21, p. 37)

A draft version of the compact was produced in late July 2020 and included “immediate measures to enable the re-opening of liquor sales on a restricted basis” (Draft Liquor Social Compact, version 2, 29th July 2020), subject to specific conditions. Industry commitments of non-specified amounts to fund partnerships with government, community-based organisations and NGOs, and to establish a “Covid-19 hospital support facility” were also noted in the same document.

The Bill was referenced at times as part of discussions regarding the social compact. For example, during a RRTT on the liquor industry meeting on 24th August 2020 (after the second temporary alcohol sales ban had been lifted) it was agreed that the Bill would be referenced within the social compact under medium term planning. Yet in September 2020, industry representatives noted that:

Anything related to policy changes must be addressed by rule of law to the relevant piece of legislation not through a Social Compact process.

(RRTT on the liquor industry meeting minutes, 7th September 2020)

At the same meeting, the Chair (a NEDLAC official) “undertook to come up with self-regulation measures together with business that go beyond written guidelines and recommended drinking limit”. Industry suggested such measures could be implemented through Aware. When labour asked for detail about the organisation, industry confirmed:

The money that is contributed [to Aware] comes from the liquor industry and is run independently through a CEO and the Board. The alcohol industry appoints the CEO and the Board.

(RRTT on the liquor industry meeting minutes, 7th September 2020)

In this and other discussions about the social compact, industry repeatedly shared various donations and investments made during the pandemic. In June 2021, a NEDLAC official shared a letter from industry directed to the President announcing a South African Breweries R2 billion investment to support economic recovery. Occasionally, however, it was reported that investments would not be followed through. For example, in late August 2020, government noted that:

There’s been a threat from SAB [South African Breweries] to withdraw the 2.5 million that they were going to invest in the sector on the basis of the fact that they were not able to trade. Therefore, seeing that the ban has been lifted, there is a need for a commitment from the sector that they will relook at this investment call.

(NRRTT on the liquor industry meeting minutes, 24th August 2020)

Meeting minutes report that industry declined to rescind the withdrawal of the funds and confirmed instead that the 2021 investment was under strict review. The result of the 2021 investment review and whether the withdrawal of funds was later rescinded is unclear.

At least seven versions of the social compact were produced and launches planned for November and December 2020, with industry suggesting inviting the President in an RRTT meeting in December. In the same meeting, however, the focus appeared to move towards shorter term measures:

Ways forward…the focus should shift from signing the compact but what can be done to prevent the resurgence.

(NRTT on the liquor industry meeting minutes, 2nd December 2020)

In 2021, South African Breweries began a legal challenge against the alcohol sales bans, which was dismissed in May 2022 [61]. This legal action was reported by NEDLAC as a key reason why the social compact had not been agreed:

By the end of the period under review, the contents of the social compact had not been agreed as one of the key participants, the South African Breweries (SAB), had requested to be excused from engagements on the matter due to its pending court case on the liquor ban issue.

(NEDLAC annual report 2020/21, p. 37)

In 2023, NEDLAC confirmed that the attempt to agree a social compact had been unsuccessful [62 p21].

## Discussion

We identified activity by 14 key alcohol industry organisations related to the Bill between 2016 and 2022. Alongside industry membership of the NEDLAC Executive Council and Trade and Industry Chamber, industry representation on five key committees facilitated their involvement in Bill-related discussions. The industry activity we report is entirely legal, with organised industry involvement written into the NEDLAC constitution [63]. This form of what is in our view very early ‘regulatory capture’ [5] gave industry a voice not only within NEDLAC, but also facilitated access to other government departments. Although ‘community’ are named as a key actor within NEDLAC, they appeared to have much less representation than other groups in the committees we identified and we found no evidence to suggest they were involved in the 2017 NEDLAC report on the Bill.

A previous study outlined how the Covid-19 pandemic provided opportunities for innovation and the prioritisation of public health in alcohol policymaking in the country [55]. Yet, it also led to industry legal threats and use of the media to oppose temporary alcohol restrictions, described more widely as signalling virtue, but promoting harm [64]. Our study adds how the crisis was also used to influence longer-term legislation, such as the Bill. Our analysis finds that the crisis interrupted the Bill’s progression, and during this interruption, industry used a combination of legal threats and donations (and at least one threat of withdrawing donations) to put pressure on the government to move away from evidence-informed legislation to self-regulation. Industry’s involvement in NEDLAC therefore enabled them to express both coercive and appeasing power [65] to attempt to shape what the social compact would involve. The promotion of what are known to be less effective, individual-based solutions are predictable and also reflect the activity of transnational corporations globally [5].

Similarly, the strategies used to frame the Bill as an unacceptable, ‘bad’ solution and less effective, individual-based responses as acceptable, ‘good’ solutions are part of the playbook used by health-harming industries globally to prevent policies that will reduce consumption and thus profits [5]. Applied to South Africa, there was an emphasis on the negative impact on small businesses and black entrepreneurs, despite the 2022 industry-funded impact assessment estimating that the biggest five companies (South African Breweries/AB InBev, Distell, Heineken, Molson Coors and Diageo) hold between 85% (by value) and 90% (by volume) of the South African market (47 p.19). That one of the ‘good’ solutions proposed was for spatial planners to avoid building schools near alcohol-licensed premises is an example of just how far apart public health and industry interests can be.

Our findings echo the industry activity reported by Bertscher et al. [10] related to the 2013 Draft Control of Marketing of Alcoholic Beverages Bill that has also not progressed through the legislature. Whereas Bertscher et al. studied the whole policy formulation process, our focus was a more detailed look at the specific mechanisms used to influence policy to align with commercial interests within a specific government body. As Bertscher et al. conclude, many of the strategies used by industry reflect global activity by health-harming industries For example, the use of a parallel, ‘grey’ pseudo-scientific literature that does not adhere to scientific norms is well-documented by alcohol and other industries globally [5, 8]. Here, industry funded two further assessments of the Bill, despite an independent socio-economic impact assessment having already been completed [53]. The first industry-funded report concluded that the Bill would have public health benefits with generally minimal economic costs. Their second document was more critical, despite concluding that two of the proposals within the Bill would save double the number of lives estimated in 2017. Further, this estimate was only based on lives saved from a reduction in road traffic incidents, rather than calculating the impact of a reduction in aggregate consumption on high rates of alcohol-related harms generally in the country, for example on rates of interpersonal violence, sexually transmitted diseases [15], and fetal alcohol spectrum disorders [66]. Industry ‘responsible drinking’ advertisements were presented uncritically in the report, despite evidence that this is a strategically ambiguous term that can reinforce existing drinking harmful attitudes and behaviours [67, 68].

Concerns regarding how government-led impact assessments are used in South Africa [10, 69] and more globally [70] centre around corporate influence undermining the development of public health policies. It was industry’s role within NEDLAC that facilitated the creation of the 2017 impact assessment, because industry had the opportunity to (a) argue that the independent socio-economic assessment that had already been done was incomplete because it had not covered potential job losses, (b) fund the study, and c) suggest the consultancy firm chosen to carry out the study. A further assessment was completed in 2022, which again was funded by industry. This evidence suggests corporate influence over impact assessment continues in South Africa via the mechanism of industry involvement in NEDLAC.

There are several limitations to our study. We focused on industry activity; we do not, therefore provide insight into the perspectives and inputs of other groups, including government, labour, and community. Previous study has found that, whilst industry is a central actor in alcohol policy formulation in South Africa, other actors play key roles, including using evidence strategically [10]. This is therefore an account of industry involvement in the Bill, rather than the policy formulation process overall, and contributions from other parties may also have delayed or otherwise influenced the Bill’s progress. For example, we found industry membership of five committees where the Bill was discussed between 2017 and 21, but other committees without industry involvement may also have been established during that time. We received only five documents related to 2017, and no documents covering 2018, 2019, and Jan-Jun 2020. We do not know the reason for these gaps, and they may mean our analysis has missed significant events that contributed to delays to the Bill. Our searches of the 2016/17, 2017/18, 2018/19, 2019/2020, 21/22 and 22/23 NEDLAC annual reports for ‘alcohol’ and ‘liquor’ also did not find any results. Yet, as outlined above, Bill-related discussions did take place within NEDLAC during that period. Increased transparency about the extent and nature of alcohol policy-related activity would help the wider community understand why the Bill has not yet progressed through the legislative process. We did not have the resources to conduct a comprehensive analysis of the two industry-funded socio-economic assessments of the Bill, and instead focused on key conclusions and industry activity related to the reports. This means we may have missed more subtle industry interventions in science related to the Bill. Although we have attempted to verify and extend the data using external sources, this was not exhaustive and there may be other publicly available documents that may shine further light on delays to the Bill. Consequently, as noted by other researchers utilising Freedom of Information legislation [31], this should be viewed as a partial account of industry activity over a limited time period, rather than a full account of why the Bill has not progressed through the legislative process. Interviews with actors involved in the Bill may also provide additional insights, as in previous study [10], although we note that the issues are sensitive and therefore any such studies should look at all actor involvement in the Bill, not just industry.

Freedom of information legislation is underutilised as a research tool [33] and there exists little guidance on how to apply the method to health policy research. Recent studies using the method have been based on UK or US legislation [34, 71–73] with reports that access has become increasingly challenging [33, 74]. Our experience mirrors this to an extent, but the quick response and access to a relatively large dataset is encouraging and indicates that the method should not be restricted to use in high-income countries. This method supported the study of less visible, appeasing power [65] and relationships between industry and other groups, as well as adding how monetary promises as well as legal threats are used by industry ‘behind the scenes’, as well as more publicly [55]. There is a need for health policy researchers and advocacy organisations to share expertise so these methods can be used in LMICs where relevant freedom of information legislation exists, mindful of the challenges unique to each country in terms of access and implementation [75].

Within the field of commercial determinants of health it is acknowledged that commercial activity can have both positive and negative health impacts [6, 76]. In South Africa, the alcohol industry has an economic role, and also either claimed to support various health and social services during the pandemic, or suggested providing such services, as part of corporate social responsibility efforts. Yet, corporate social responsibility initiatives more widely have been found to be largely ineffective at addressing alcohol and other harms [5, 77], and the cost of alcohol harms in South Africa is high [20]. The evidence is clear on what intervention is needed to address alcohol harms in the country [78]; the lack of progress has therefore been described as a result of a lack of political will to implement changes that might reduce profits [79]. Our findings add nuance to this perspective, because involvement in NEDLAC as a social partner gave industry the opportunity to attempt to influence the Bill. Which groups are included in government processes and what role they play could therefore be an important factor that could influence the will (and action) for change. Our findings also mirror previous studies on corporate influence in nutrition and alcohol policy in the country [10, 11]. Just as alcohol is no ordinary commodity [80], the industry is no ordinary stakeholder in matters related to public health. Industry’s role in impact assessments and public health policy formulation should be reconsidered globally, and it should be viewed as a (largely transnational) commercial actor, rather than the social partner it is described as within NEDLAC locally.

## Data Availability

Where already published online, data have been cited accordingly. Resources were not available to anonymise all names and other personal information about individuals in the data, which is a requirement under South African law to publish data received via a Request for Access to Record.

## Footnotes

1 The B-BEEE ACT establishes a legislative framework for the promotion of black economic empowerment with the expressed intention to address the legacy of apartheid and promote participation of black people in the economy.
